# Exploring biased attention towards body-related stimuli and its relationship with body awareness

**DOI:** 10.1038/s41598-017-17528-2

**Published:** 2017-12-08

**Authors:** Gerardo Salvato, Gabriele De Maio, Gabriella Bottini

**Affiliations:** 1 Cognitive Neuropsychology Centre, ASST “Grande Ospedale Metropolitano” Niguarda Hospital, Milano, Italy; 2 Department of Brain and Behavioural Sciences, University of Pavia, Pavia, Italy; 3 NeuroMI, Milan Centre for Neuroscience, Milan, Italy

## Abstract

Stimuli of great social relevance exogenously capture attention. Here we explored the impact of body-related stimuli on endogenous attention. Additionally, we investigate the influence of internal states on biased attention towards this class of stimuli. Participants were presented with a body, face, or chair cue to hold in memory (Memory task) or to merely attend (Priming task) and, subsequently, they were asked to find a circle in an unrelated visual search task. In the valid condition, the circle was flanked by the cue. In the invalid condition, the pre-cued picture re-appeared flanking the distracter. In the neutral condition, the cue item did not re-appear in the search display. We found that although bodies and faces benefited from a general faster visual processing compared to chairs, holding them in memory did not produce any additional advantage on attention compared to when they are merely attended. Furthermore, face cues generated larger orienting effect compared to body and chairs cues in both Memory and Priming task. Importantly, results showed that individual sensitivity to internal bodily responses predicted the magnitude of the memory-based orienting of attention to bodies, shedding new light on the relationship between body awareness and visuo-spatial attention.

## Introduction

Visuo spatial attention tunes behavioural and neural processing in order to select relevant stimuli among others within the environment^1^. Two main sources of modulatory bias have been generally recognized: exogenous and endogenous shift of attention. Visuo-spatial attention could be exogenously captured by the physical salience of certain stimuli present in the environment. For instance, stimuli of great social and biological relevance would engage and hold more attention than non-biological stimuli^2–6^. Ro *et al*.^2^ have shown that the detection of change in a circular visual array composed by 8 different images, was faster when the variation involved faces. As in the case of faces, body pictures may be prioritized for attentional selection. It has been shown that body pictures benefit from a faster visuo-spatial processing compared to non-body stimuli. In a modified attentional capture visual search paradigm, a faster and stronger processing of body parts compared to cars, food, instruments, and plants has been found^7^.

In endogenous shift, visuo-spatial attention is attracted towards stimuli matching the content of memory, such as a certain location in space^8,9^, or a certain stimulus^10,11^. These representations become a “search template” (or “attentional set”) provided by memory, which may bias attention diminishing or increasing reaction times (RTs) in visual search^10,12–14^. For instance, it has been shown that working memory (WM) may bias selection, independently of behavioural goals, leading to the capture of attention to irrelevant stimuli matching the WM template^10,12^. In traditional laboratory paradigm exploring such effect^11,15–18^, participants are firstly required to memorize an item, usually an object, or to just attend to it. After a short interval, they are asked to perform an unrelated visual search task, in which a particular feature has to be selected over a number of stimuli. The search array could include the cue item flanking the target (valid condition), the distractor (invalid condition), or the cue could be absent from the search array (neutral condition). Consistently, results have shown faster RTs in valid compared to neutral trials, with slower RTs in invalid compared to neutral trials, demonstrating that the item memorized affects attentive performance^11,15^. The guidance effect is weaker or absent when the first target is required to be merely attended.

Although previous research indicates that social stimuli exert an exogenous shift of attention, less is known about the endogenous effects from WM on attention. Bodies and faces are especially salient stimuli due to their implication in social cognition, as both contribute to the recognition of other people and the identification of their age, gender, intentions, and emotional state. Thus, holding in WM such stimulus categories may be particularly effective at capturing visual attention, because salient items have privileged access to WM^19^. To test this hypothesis, we administered thirty-three healthy participants with a previously published memory-based attention paradigm^17,18^, varying the nature of the stimuli with bodies, chairs, and faces. We hypothesized that if social stimuli exogenously shift visuo-spatial attention, there should be an enhancement of the attentional bias towards them when they are also held in memory.

Furthermore, it has been recently demonstrated that body signals may actively influence visual consciousness^20,21^. For instance, Solomon and colleagues^21^ have shown that the congruence of real and viewed hand position influences the formation of visual consciousness even when it is task irrelevant. In a perceptual suppression paradigm, participants were required to judge the orientation of a stimulus embedded in a task-irrelevant picture of the hand. Results have demonstrated that the perceptual suppression was broken more rapidly when the position of the hand picture was congruent with the position of the participant’s hand. Here, we aimed at exploring whether body signals influence the extent to which attentional resources are biased by body-related images. To this aim, we collected a subjective index of body awareness measured by means of the Body Perception Questionnaire (BPQ)^22^. We hypothesized that if bodily state influences perception, the level of body awareness would modulate the biased orienting of attention for body-related stimuli.

## Results

### Accuracy

In the Memory and Priming tasks, participants were overall highly accurate at the visual search (Body cues (*M* = 0.97; *SE* = 005), Face cues (*M* = 0.97; *SE* = 0.004), Chair cues (*M* = 0.96; *SE* = 0.006). Errors were minimal and were not analysed further.

### RTs

Incorrect responses and RTs that were ± 3 standard deviations from the mean were removed (body cue trials: 3.6%; face cue trials: 3.5%; chair cue trials: 3.5%). Catch trials from the Memory task were also removed to equate the number of trials between the tasks. A repeated measures ANOVA was performed with Task (Memory, Priming), Stimulus (Body, Chair, Face), and Validity (Valid, Neutral, Invalid) as within-subjects factors. RTs were insert as dependent variable.

**Figure 1:**
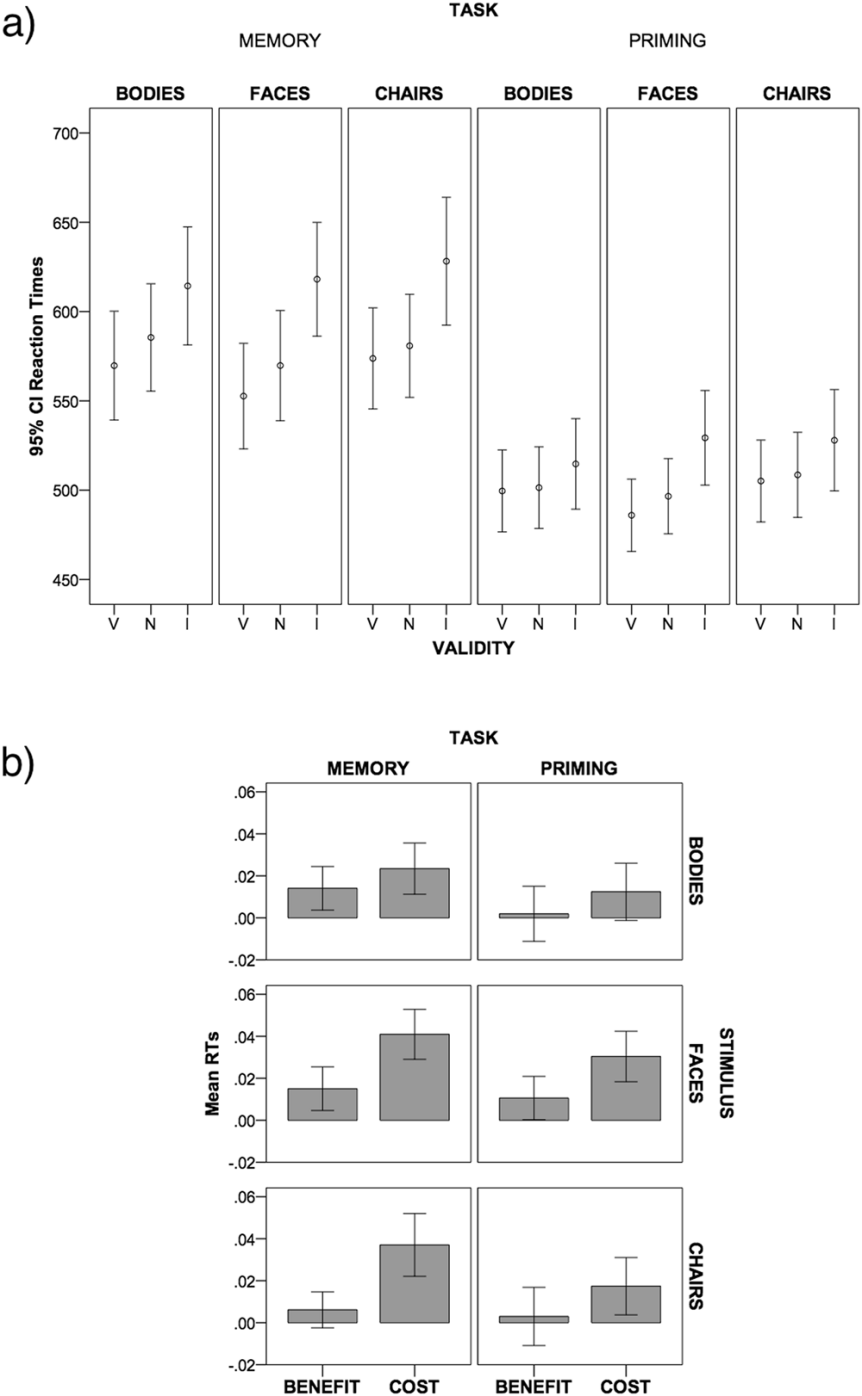
Results. Panel (a) *95%* Confidence Interval of mean reaction times in the visual search phase for the Priming and Memory task. The cue (hold in memory or just attended) flanked the target in the visual search (Valid condition, V), flanked the distractor (Invalid condition, I) or was absent from the array in the visual search (neutral condition, N). Panel (b) The graph shows mean RTs (error bars: 95% Confidence Interval) of benefit (neutral trials – valid trials) and cost (invalid trials – neutral trials) in visual search as a result of holding a cue in memory (Memory task) or just attending at it (Priming task).

Results showed that participants performed the two tasks differently. We found a main effect of Task (F_(1,32)_ = 59.9; *p* < 0.001; η^2^_p_ = 0.7). Participants were slower in the Memory (*M* = 588.1; *SE* = 14.6) compared to the Priming task (*M* = 507.7; *SE* = 10.9) (see Fig. 1).

Participants differently processed the three stimulus categories regardless the nature of the task. Results showed a main effect of Stimulus (F_(2,64)_ = 12.9; *p* < 0.001; η^2^_p_ = 0.3). Post-hoc Bonferroni-corrected pairwise comparisons showed an overall RTs advantages for faces (*M* = 542.5; *SE* = 11.6) compared to chairs (*M* = 554.5; *SE* = 11.9) (*p* < 0.001) and bodies (*M* = 547.5; *SE* = 11.9) (*p* = 0.028), and for bodies compared to chairs (*p* = 0.025). It is important to note that differences in the search task RTs between chair, face, and body pictures could be related to differences in memorization difficulty between these stimulus categories. In order to explore this possibility, we performed a repeated measures ANOVA with Stimulus (Body, Face, Chair) as within-subjects factor and accuracy at the recognition task (Memory task catch trials) as dependent variable. Results showed that there was no difference in memory accuracy between stimulus categories ((*F*_(2,64)_ = 0.7; *p* = 0.474; *η* ^2^_*p*_ = 0.02) (body pictures: *M* = 0.95; *SE* = 011, face pictures: *M* = 0.96; *SE* = 0.008, chair pictures: *M* = 0.96; *SE* = 0.009). To investigate for possible differences in RTs between stimulus categories at the recognition task, we also performed a repeated measures ANOVA with Stimulus (Body, Face, Chair) as within-subjects factor and RTs at the recognition task (Memory task catch trials) as dependent variable. Results showed no differences in RTs between body (*M* = 1013 ms; *SE* = 32.8), face (*M* = 1030 ms; *SE* = 37.4), and chair pictures (*M* = 1023 ms; *SE* = 34.1) (*F*_(2,64)_ = 0.6; *p* = 0.546; *η* ^2^_*p*_ = 0.02) at the recognition task.

The typical cost and benefit effect resulting from the influence of memory on attention was present. Results showed a main effect of Validity (F_(2,64)_ = 60.1; *p* < 0.001; η^2^_p_ = 0.7). Bonferroni-corrected pairwise comparisons showed that participants were faster in the valid compared to neutral (*p* = 0.008) and invalid trials (*p* < 0.001). Furthermore, invalid trials were slower than neutral trials (*p* < 0.001).

Participants’ performance in the visual search benefited from the cue held in memory, in the Memory task only. An interaction Task by Validity was present (F_(2,64)_ = 10.1; *p* < 0.001; η^2^_p_ = 0.2). Further analyses showed that in the Memory task valid trials were faster compared to neutral trials (*p* = 0.004). Invalid trials were slower compared to neutral (*p* < 0.001) and valid trials (*p* < 0.001). Conversely, the beneficial effect was not present in the Priming task. There was no difference between valid and neutral trials (*p* = 0.723), although invalid were slower compared to valid (*p* < 0.001) and neutral trials (*p* < 0.001).

The Stimulus by Validity interaction was also significant (F_(4,128)_ = 5.7; *p* < 0.001; η^2^_p_ = 0.2). To better frame this interaction we performed three repeated measures ANOVAs with Validity (Valid, Neutral, Invalid) as within-subjects factor for each stimulus category. In particular, the three stimulus categories differed in terms of RTs benefit in priming and holding a cue in memory. We found a significant main effect of Validity for each stimulus category (body:(*F*_(1,32)_ = 21.5; *p* < 0.001; η^2^_p_ = 0.4); face:(*F*_(1,32)_ = 68.9; *p* < 0.001; η^2^_p_ = 0.7); chair:(*F*_(1,32)_ = 27.1; *p* < 0.001; η^2^_p_ = 0.5)). Bonferroni-corrected pairwise comparisons showed that in case of body pictures, there was a trend for valid trials being faster than neutral trials (*p* = 0.058). Valid and neutral trials were faster than invalid trials (both comparisons *p* < 0.001). With face cues, valid trials were faster than neutral and invalid trials (valid *vs* neutral *p* = 0.002; valid *vs* invalid *p* < 0.001). Invalid were slower than neutral trials (*p* < 0.001). In case of chair cues, we found no differences between valid and neutral trials (*p* = 0.591), whereas valid and neutral trials were faster than invalid trials (both comparisons *p* < 0.001).

The interactions Task by Stimulus (*F*_(2,64)_ = 1.7; *p* = 0.194; η^2^_p_ = 0.05), and Task by Stimulus by Validity (*F*_(4,128)_ = 0.7; *p* = 0.748; η^2^_p_ = 0.01) were not significant.

### Memory-based orienting of attention

To ensure that the different response speeds (Memory/Priming tasks) did not mask any qualitative difference between stimuli in the biasing attention effect, we also calculated normalized measures of the validity effect (orienting effect magnitude) [(invalid -valid)/(invalid + valid)]^9,23^ for each stimulus category, in both tasks.

**Figure 2:**
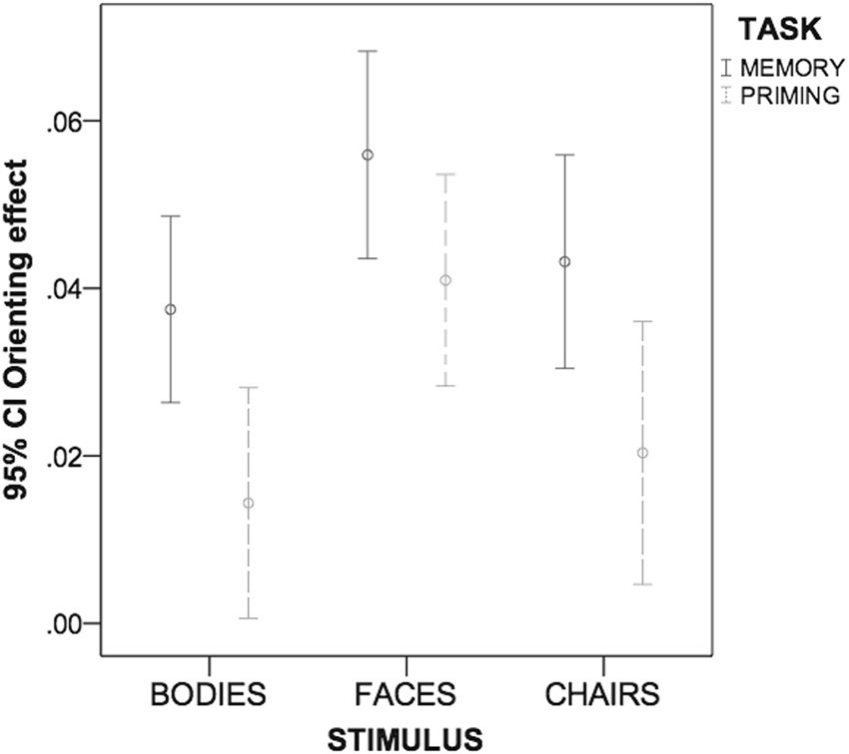
The graph shows mean (95% confidence intervals) of the normalized validity cost mean (orienting effect) of body, face and chair cues in the Memory (solid bars) and Priming task (dashed bars).

We performed a repeated measures ANOVA with Task (Memory, Priming) and Stimulus (Body, Chair, Face) as within-subjects factors. The magnitude of orienting effect was insert as dependent variable. Results showed a larger orienting effect in the Memory task, and faces showed the biggest magnitude compared to other stimuli (see Fig. 2). We found main effect of Task (*F*_(1,32)_ = 14.7; *p* = 0.001; η^2^_p_ = 0.3), indicating a greater magnitude for the orienting effect in the Memory task (*M* = 0.46; *SE* = 0.004) compared to the Priming task (*M* = 0.25; *SE* = 0.005). There was a main effect of Stimulus (*F*_(2,64)_ = 9.6; *p* < 0.001; η^2^_p_ = 0.2). Bonferroni-corrected post-hoc pairwise comparisons showed an overall advantage for faces (*M* = 0.48; *SE* = 0.004) compared to chairs (*M* = 0.32; *SE* = 0.006) (*p* < 0.001) and bodies (*M* = 0.26; *SE* = 0.005) (*p* = 0.010). There was no difference in the orienting effect for bodies compared to chairs (*p* = 0.890). The interaction Task by Stimulus was not significant (*F*_(2,64)_ = 0.3; *p* = 0.712; η^2^_p_ = 0.1).

### Relationship between internal states and orienting effect

To explore the hypothesis that internal states influence the biased orienting of attention for body-related stimuli, we modelled a multivariate multiple regression using the orienting effect scores of the body, face and chair cues resulting from the Memory and Priming tasks as dependent variables, and the body awareness scores as covariate (predictor). To deal with outliers affecting the values of the estimated regression coefficients, we also performed casewise diagnostic on standardize residuals. We excluded from the subsequent analyses one outlier (outside ± 3 standard deviations) (*SD residual*: 3.1). Results showed that the body awareness scores selectively predicted the orienting effect magnitude of body cues at the Memory task (*b* = 0.53, *t*_(31)_ = 3.5, *p* = 0.002; *R* ^2^ = 0.28, *F*_(1,31)_ = 11.9, *p* = 0.002). In other words, participants with higher level of body awareness showed larger orienting effect for bodies at the Memory task only. This relation was absent for other stimulus categories. To implement the frequentist statistical analyses, we performed a Bayesian linear regression^24^ to test whether there was evidence for supporting the alternative hypothesis against the null hypothesis. We found strong evidence for the alternative against the null hypothesis (BF_10_ = 20.8).

## Discussion

In line with previous findings, our results demonstrated that holding a cue in WM implied a behavioural cost, resulting in slower RTs in the Memory compared to the Priming task^10,11,15,25^. Recent findings have also shown that the behavioural difference between these two tasks is paralleled by the engagement of different neural networks. In an fMRI study adopting a similar paradigm (geometrical shapes instead of real pictures), holding a cue in memory that reappeared in the visual search task, activated the superior frontal gyrus, mid-temporal and occipital areas. Conversely, the mere repetition of the cue also reoccurring in the visual search induced de-activation in the same regions^17^.

We also showed that in the Memory task, valid trials were faster than neutral trials, whereas they were equal in the Priming task. One might hypothesize that WM content induced a benefit on RTs automatically biasing attention towards the memorized cue. Alternatively, our findings could reflect the fact that participants have tried to refresh their memory trace when the WM cue reappeared in the visual search task, deliberately attending to represented WM cues (strategic resampling account^26^). Although there is evidence in literature discarding the latter account^27–29^, the present study is not able to rule it out. Further specific studies are needed to better disentangle this issue.

Interestingly, here we found that the validity effect was regulated by the stimulus category. Both body-related cues showed enhanced visual search when they reappeared in the same position of the target. Chair cues did not showed such boosting of attention, as demonstrate by equal RTs for valid and neutral trials. This pattern was equally present in the Priming and in the Memory task.

Although the nature of the stimulus to be processed equivalently affected the Memory and the Priming task, when body and face cues were simply presented, or required to be memorized, reaction times were faster compared to chair cues. The visual processing advantage for biologically relevant stimuli is adaptive to the evolution of humans. In everyday life, we constantly cooperate with others, and for instance, we need to readily find and recognize the person we are going to interact with amongst other people. To this aim, we use information such as body shape and face. One might hypothesize that the behavioural top-down and bottom-up visual attention preference for body-related cues could be also explained from the cortical modularity of this type of stimuli. A large body of evidence has shown that human brain represents the body in a domain-specific manner, within selective specialized neural networks^5,30–33^. Imaging studies have demonstrated that specific brain areas, such as the Extrastriate Body Area (EBA)^32^ are causally involved in creating and/or maintaining a precise representation of the body but not of object shapes. Furthermore, visual perception of faces selectively activates a region of the occipital cortex identified as Fusiform Face Area (FFA). Importantly, the FFA would also be involved in the face identification process^34^. Further supporting the modular account in which category-selective brain areas contribute to discrimination of their preferred categories, a triple dissociation has been observed between the visual processing of bodies, faces and objects. It has been shown that transcranial magnetic stimulation (TMS) over right Occipital Face Area modulated discrimination of faces but not objects or bodies; TMS over right EBA modulated discrimination of bodies but not faces or objects; TMS over right Lateral Occipital Area impaired discrimination of objects but not faces or bodies^35^.

Here we also found that face cues showed a larger orienting effect compared to other stimulus categories in both the Memory and Priming task. Moreover, body and chairs elicited the same magnitude of priming and WM-based orienting effect. This finding may reflect our expertise for faces, and the importance that faces play in social cognition^36^. As humans, we are regularly exposed to faces, and we focus our attention to faces more than bodies in order to interact, communicate, and understand emotions. Furthermore, it has recently been demonstrated that the cortical modularity for face perception is shaped by experience and age^37^, suggesting an adaptive mutual body-environment influence, which tunes our brain and behaviour.

The present study provided novel evidence on the role of representation of visceral responses accessible to awareness, which modulates the way we perceive the environment. We found that higher awareness for internal bodily responses resulted in a larger orienting effect generated from body cues when participants explicitly memorized them. Research concerning the role of interoceptive signals on cognition is scarce. Nevertheless, some evidence is present^20,21,38^. In a recent study by Ronchi *et al*.^38^, it has demonstrated that interoception enhanced visual processing for body images when shown to the participants in synchrony with their heartbeat. Our findings suggest a tighter link than previously hypothesized between the self-perception and memory-based attention. Although the neuro-functional bridge between the two remains to be investigated, one might hypothesize that the “body matrix” could represent a good candidate. The body matrix has been identified as a holistic representation of the body, involving multisensory, spatial processing, and homeostatic signals^39^. It has been postulated that the connection between the insula and the parietal cortex may represent the neural substrate of the body matrix^39^. The insular cortex has been identified as a central area for the representation of the body in the brain^40^, and may be a crucial region for integrating internal and external stimuli. It is also worth noting that the BPQ awareness subscale correlates with grey matter volume in the right anterior insula^41^. Furthermore, the parietal cortex is known to be strongly involved in visuo-spatial processing, and it has been recently demonstrated that the grey matter volume of this area correlates with the ability to use the memory content to facilitate visual search^42^. Additionally, patients with parietal brain damage suffering from visual extinction, can show enhanced awareness for contralesional targets when they match the contents of memory^43^.

In summary, we found no additional advantages in holding a biologically relevant cue in WM during visual search. Furthermore, body and face cues benefited from their re-appearance flanking the target in visual search (RTs valid trials < neutral trials) in both, Priming and Memory tasks. Face cues showed a general larger attentional bias compared to body and chair cues. Notably, the subjective level of body awareness predicts the magnitude of the WM-based orienting of attention towards bodies.

## Materials and Methods

### Sample size calculation

We used a modified version of a previous published experimental paradigm^18^, which has been used to measure memory-based facilitation for biologically relevant target detection/discrimination. For this reason, we estimated the group size needed to show a difference between diverse types of stimuli (Bodies, Faces, Chair) on the basis of this study. The authors have found a significant advantage for food versus non-food stimuli (%RT for [non-food minus food]/nonfood) for valid trials in the Memory task compared to the Priming task (4.2 ± 3.4 vs. 1.9 ± 4.3%, *p* < 0.05). In the present study, we hypothesized that the body-related cues used would show a larger memory-based attentional benefit than non-body related cues, with an *alpha* = 0.05 on a dependent means one-tailed t-test. Using a freely available sample-size calculating tool (G*Power), the suggested sample size was 33 participants.

### Participants

Thirty-tree right-handed healthy adults (16 males, 17 females; ages range 20–39, *M* = 27.6, *SD* = 4.8; years of education *M* = 16.4, *SD* = 2.6) participated. All were native Italian speakers, had normal or corrected-to-normal vision, and had no previous history of mental or neurological illness. In accordance with the Declaration of Helsinki (BMJ 1991; 302: 1194), all the experimental procedures were approved by the Ethical Committee of the Department of Brain and Behavioral Sciences, University of Pavia. Informed consent was obtained prior to participation in the experiment.

### Task and procedure

#### Stimuli

Black and white digital photographs of bodies, faces, and chairs were used to construct the visual stimuli. Eight body pictures (4 males, 4 females) were selected from the BEAST database (neutral body postures)^44^. Eight face pictures with neutral emotional expression (4 males, 4 females) were selected from the Ekman and Friesen series^45^. Eight chair pictures were selected from the EBA localizer picture database^32^. The images were sized to fit within box against a grey background (12,1° × 12,1° of visual angle at a viewing distance of 60 cm). Pictures were equated for luminance within and between stimulus categories (luminance value: 150). Two black geometrical figures, a circle and a square (2,5° × 2,5° of visual angle at a viewing distance of 60 cm), were used as search targets.

#### Tasks

**Figure 3:**
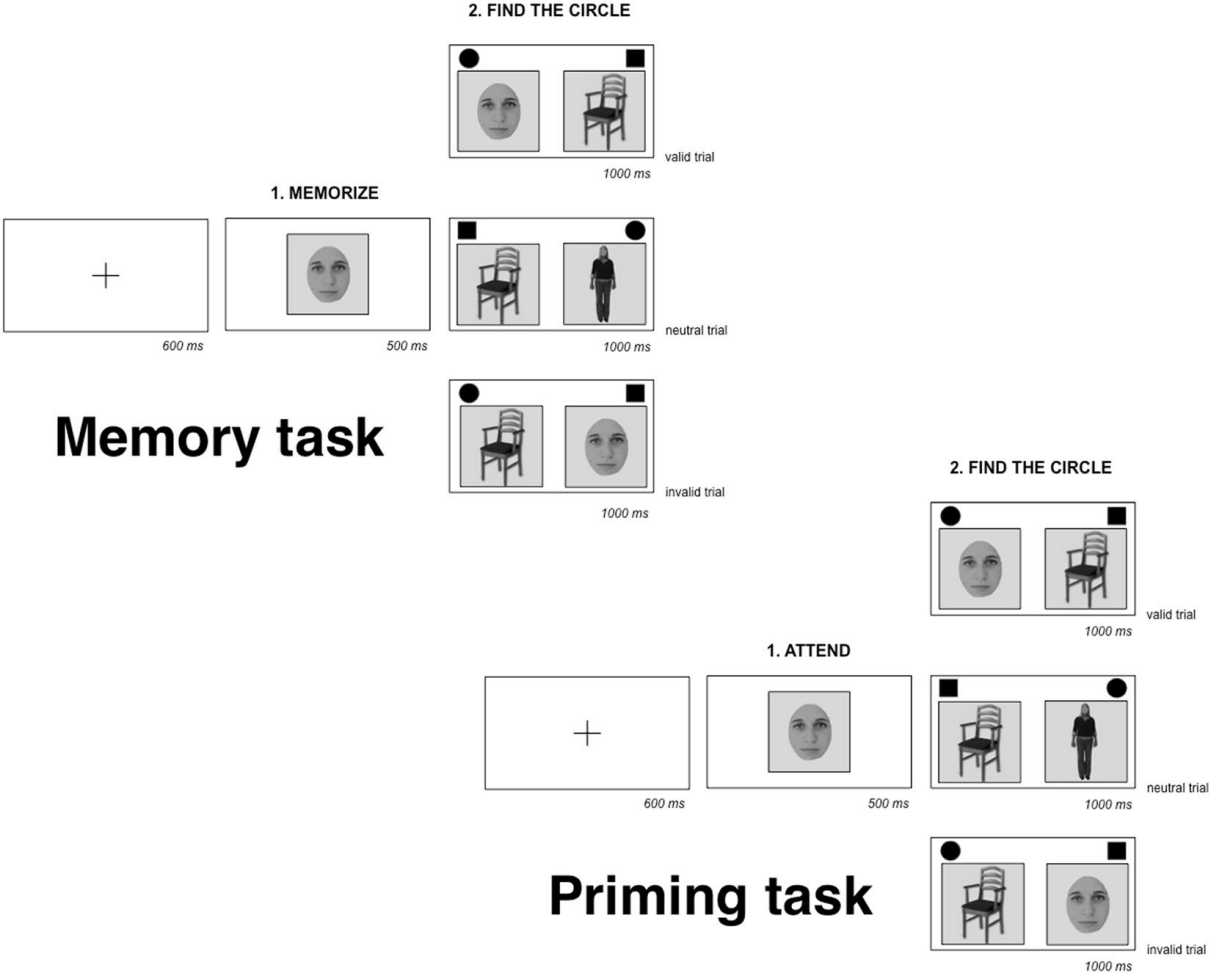
Example of the Memory and Priming task. The face shown in this picture is for display purpose only. In the experiment, we used faces from the Pictures of Facial Affect (POFA) database^45^. The chair in the figure was retrieved from the EBA localized dataset^32^ (http://pages.bangor.ac.uk/~pss811/page7/page7.html). The body is part of the Bodily Expressive Action Stimulus Test (BEAST) database^44^ (http://www.beatricedegelder.com/beast.html). Size of stimuli and distances between them are here schematically presented.

The experiment included two tasks: Memory and Priming (see Fig. 3). In the Memory task the trial started with a central fixation cross appearing on the screen for 600 ms. Soon after, a body, face, or chair picture appeared centrally on the screen for 500 ms. Participants were explicitly required to hold in memory that picture throughout the trial. After the memory cue (250 ms), participants were presented with a search array consisting of a circle and a square appeared together on the left and right side of the screen. Both stimuli had equal probability to appear on the two sides. Participants were required to find the circle, by pressing the left or the right arrow on the computer keyboard within a 1000 ms time window. A body, face, or chair picture flanked the distractor and the target at a 0.5 cm distance. The two images were presented at the centre of the screen at a 1 cm distance between each other. The visual search consisted of three conditions occurring randomly with equal probability: (i) on valid trials, the circle was flanked by a picture that was identical to the cue, and the distractor in the search display was flanked by a picture from one of the other stimulus categories, (ii) on invalid trials, the square was flanked by a picture that was identical to the cue, and the circle was flanked by a picture from one of the other stimulus categories, (iii) on neutral trials both the square and circle were flanked by pictures from categories different from the memory cue. In order to ensure that participants had memorized the cue, in 20% of trials, after the visual search, a body, face, or chair picture appeared at the centre of the screen. The image could be identical to the one memorized or from a different category. Participants were asked to indicate if the picture was equal or different compared to the one they were holding in memory within a 3000 ms time window. They pressed on the computer keyboard the letters “S” (same) if the stimulus matched the memory cue or “D” (different) if the cue and the stimulus to be remembered did not match.

The Priming task was similar to the Memory task, except from the instruction regarding the first cue. After a central fixation cross (600 ms), a body, face, or chair picture appeared at the centre of the screen for 500 ms. While in the Memory task, we asked participants to hold in memory the first cue, in the Priming task participants were required to just pay attention to it. For this reason, in the Priming task, catch trials were designed to ensure that participants had paid attention to the cue. In 20% of trials after the fixation cross, a cue appeared on the screen for 500 ms and followed by another cue from a different stimulus category (500 ms). When the initial cue changed, participants were required to hold their response in the following visual search array. The subsequent visual search phase of the Priming task was designed as in the Memory task.

The Memory and the Priming tasks consisted of 300 trials each, included 12 initial practice trials that allowed participants to familiarize with the experiment. Participants completed the tasks in a counterbalanced order.

#### Body Awareness

Between the Memory and Priming task, participants were administered with the Body Perception Questionnaire (BPQ). The BPQ is a self-report 122-item questionnaire assessing body awareness, stress response, autonomic nervous system reactivity, and stress style^22^. The body awareness subscale incorporates bodily sensations (e.g., “During most situations I am aware of: Swallowing frequently; A ringing in my ears; An urge to cough to clear my throat; My body swaying when I am standing”). The autonomic nervous system reactivity subscale contains items investigating the reactivity of cardiovascular, respiratory, digestive, and temperature regulation functions. The stress response and stress style subscales measure the participants’ awareness of bodily sensation in response to stressful situations. Participants were required to indicate their awareness of each sensation in each subscale using a five-points scale ranging from ‘never’ to ‘always’. In line with the aim of the study and with previous literature^41,46^, here we took into account only scores at the body awareness subscale.

#### Apparatus

The tasks were programmed using OpenSesame^47^ software package version 0.27.2 (http://osdoc.cogsci.nl/). A personal computer controlled the stimulus displays and collected the responses. The stimuli were displayed on a 24-inch monitor with a resolution of 1028 by 768 pixels and a 60-Hz refresh rate. The BPQ was administered in paper-and-pencil format.
